# Supplementary material for: Are carbon emissions trading and green financial instruments synergistic? -Comprehensive quantitative research based on content analysis
Source: PLoS One. 2024 Mar 7;19(3):e0298601. doi: 10.1371/journal.pone.0298601 (PMC10919601; doi:10.1371/journal.pone.0298601)
Supplement: S1 Data — (ZIP) [file pone.0298601.s001.zip › Data/地级市Z_F工作报告.zip!/─┌├╔╣┼/╬┌└╝▓∞▓╝╩╨/└┤╫╘╣1⁄2╓┌║┼í╛┬φ┐╦╩2╛▌═°í┐_2014.docx]

各位代表：

现在，我代表市人民政府向大会报告工作，请予审议，并请各位政协委员和列席会议的同志们提出意见。

一、2013年工作回顾

过去的一年，面对复杂的经济形势和繁重的发展任务，在自治区党委、政府和市委的正确领导下，在市人大和政协的监督支持下，市政府团结带领全市各族干部群众，坚持以“8337”发展思路统领全局，求真务实，开拓进取，全面推进“三个基地”、“三个中心”和“五个区”建设，圆满完成了各项目标任务。

经济发展稳中向好，综合实力不断增强。在宏观经济形势十分困难的情况下，抓住投资这个“牛鼻子”，咬定发展不动摇，坚持稳中有为、稳中求进，经济实现了持续健康发展。预计地区生产总值完成855亿元,增长9%；固定资产投资完成800亿元，增长23%；地方财政总收入完成76亿元，增长15%；规模以上工业增加值完成335亿元，增长12%；社会消费品零售总额完成236亿元，增长11%；城镇居民人均可支配收入和农牧民人均纯收入分别增长10%和15%，达到20470元和6760元。主要指标增速位居全区前列。

产业结构不断优化，运行质量明显提升。围绕自治区“五大基地”产业发展定位，出台了产业发展指导意见和10个重点产业专项规划，推动产业结构转型升级。农牧业生产喜获丰收。大力推广综合集成技术。冷凉蔬菜达到45万亩，设施蔬菜达到15万亩，高效节水灌溉面积达到280万亩。新建草莓基地2500亩，达到5000亩。马铃薯单产、总产均创历史新高。粮食总产23.7亿斤，增产6亿斤。规模化养殖水平明显提高。建成“百千万”养殖园区151处。牧业年度牲畜总头数达到801万头(只)，生猪、肉鸡出栏分别达到203万口和1312万只，畜牧业产值占农牧业总产值的比重提高10个百分点，达到62%。产业化发展步伐加快。引进大北农、正邦等一批产业化重点项目，规模以上农畜产品加工企业发展到140家，加工转化率达到48.3%，提高6个百分点。国家农业标准化示范区达10个，新增国家级专业合作示范社5个、自治区级示范社15个。品牌建设成效明显。新增“三品一标”认证产品41个，达到192个，认证面积434万亩。国家农业科技园区获得批准，跻身国家“一城两区百园”战略体系。工业经济稳步发展。面对严峻的经济下行压力，积极采取保运行、稳增长、促转型等有效措施，运用减少涉企收费、金融支持实体经济政策，稳定了工业生产和企业运行。实施电价补贴和电力多边交易，135户企业受益，降低企业用电成本6.2亿元，新增税收4.09亿元，帮助企业度过难关。全市新增工业用电量49亿千瓦时，新增用电负荷100万千瓦以上，最大用电负荷达到324万千瓦。工业经济集约化程度显著提升，六大主导产业完成增加值303亿元，占全部工业增加值的78.7%。园区工业增加值达到351亿元，占全部工业增加值的91%。丰镇氟化工园区、后旗建材化工园区和察哈尔经济技术开发区营业收入均超百亿元。现代服务业健康发展。区域性商贸物流中心建设扎实推进。“三个交易中心”实现营业收入96亿元，增长18.5%。内蒙古西北煤炭交易中心建成运行，煤炭和马铃薯国家级检验检测中心启动实施。皮革鞣制、加工、销售产业有序推进，华北地区最大的皮革皮件购物中心——集宁国际皮革城建成运营。区域性草原文化旅游度假中心建设步伐加快。“一心两翼三大境区”构架确立，旅游业固定资产投资达到20亿元，增长97%。新建旅游公路6条181公里，新增4A级景区3个。接待游客800多万人次，增长45%；实现旅游收入73亿元，增长65%。金融服务实体经济能力进一步增强。信贷规模创历史新高，预计金融机构人民币贷款余额达到432亿元，贷存比66.3%，比年初提高9个百分点。市财政投入信贷担保激励资金6000多万元，为中小微企业提供担保贷款10亿元。社会融资规模突破200亿元。县域经济和非公经济快速发展。出台指导意见和鼓励支持政策，成立领导机构专项推进。县域综合经济实力进一步增强，特色主导产业更具发展活力，非公有制经济占GDP的比重达到60%，高于全区近10个百分点。区域经济合作积极推进。深入开展京蒙对口帮扶，与天津、山西、山东、广东等地的合作不断深化。

城镇建设取得突破，人居环境显著改善。全年实施城建项目1054个，完成投资260亿元，较上年增长9个百分点。城镇化率提高到45%，城乡面貌发生可喜变化。城镇布局更加优化。中心城区城市框架不断拉大，规划面积350平方公里，建成区面积达到60平方公里，“一市两区三组团”发展格局更加清晰。各旗县市城关镇控制性详规实现全覆盖，市域村镇体系规划全部完成，新老城区、建制镇和新农村新牧区建设统筹推进。住房条件大幅改善。全市商品房竣工面积达到208万平方米，房地产市场平稳运行，住宅、商贸、产业功能分区更加合理。开工建设各类保障性住房2.8万户，完成农村牧区危房改造2.3万户，均超额完成自治区下达任务。建成互助幸福院283处，解决2.7万户农村牧区老年人的养老问题。市政设施日趋完善。全市人均道路面积和人均公园绿地面积达到自治区平均水平。中心城区编制完成供热、公交等专项规划，实现热源联网运行，新增供热能力1000多万平方米，集中供热普及率达到93%。建成公交场站7处，开通延伸公交线路13条，新增公交车112台。南绕城一级公路主线完工，110国道城区快速通道建成通车。城市景观更具特色。坚持打好“山水绿”三张牌，中心城区“三山两河”建设效果明显，成为宜居城市新名片；各旗县市建设了一批各具特色的社区公园、郊野公园、绿道绿廊。全市新建公园17个，新建城市景观带660万平方米，建成区绿化覆盖率达到22.3%。城市地更绿、路更宽、水更清，环境更加舒适靓丽，人民幸福指数明显提升。

基础设施更加完善，发展条件不断优化。坚持把生态建设摆在更加突出的位置，抓好国家项目实施，突出重点区域绿化，完成营造林221万亩，森林覆盖率达到22.7%。准兴重载等9条公路建成通车。新增高速公路368公里，总里程跃居全区第一。建成通村油路90条、1000公里，嘎查村通沥青水泥路率由44.5%提高到53%，苏木乡镇客运站覆盖率由75%提高到86%。支线机场批准立项，主体工程建设顺利，呼张客专征拆工作全面启动。建设110千伏以上输变电工程20项，新增变电容量275万千伏安。卓资隆胜等重点水库建设扎实推进，完成11座病险水库除险加固，解决了17.8万人饮水安全问题。用足用好自治区地勘扶持政策，实施地勘项目120个，萤石等47个项目取得初步成果，资源保障利用水平明显提高。

民生工作扎实有效，保障水平明显提高。民生支出占财政总支出的82%。“九件实事”全面完成。就业创业工作稳步推进。“金蓝领”培训工作成效明显，“乌兰美”家政服务成为品牌。为4000名创业者发放小额担保贷款1.66亿元。转移农牧民就业34.4万人，新增城镇就业1.69万人，城镇登记失业率控制在3.8%以内。社会保障水平不断提高。社会保险扩面任务超额完成。企业退休人员养老保险待遇提高10%，城镇职工医保、城镇居民医保、新农合市内住院实际报销比例分别达到75%、70%、70%。城乡低保、五保等特殊困难群体得到有效救助，为全市32万户低收入农牧民每户免费发放1吨取暖煤。对当年新入学的3000名农村牧区贫困大学生在校期间每人每年救助1500元。扶贫攻坚取得实效。落实省市县乡四级领导干部联系贫困点制度，投入资金12.2亿元，10.1万贫困人口稳定脱贫。

社会事业全面进步，社会管理成效显著。教育事业健康发展。“三项改革”深入推进，教学活力明显增强。义务教育和职业教育经费增速全区第一。完成校舍建设36万平方米，建成塑胶操场71个。中心城区8所学校、5所幼儿园和农村牧区100所幼儿园投入使用。基本医疗和公共卫生服务体系不断完善。自治区卫生城市复检顺利通过。市中心医院门诊楼、传染病医院和9所县级医院投入使用。低生育水平保持稳定。科技贡献率不断提升，成果转化效果明显。群众性文化活动蓬勃开展，实施“户户通”工程14.5万户。安全生产形势平稳好转。食品安全监管工作全面加强，在线监控启动运行，小餐饮、小摊贩、小作坊进一步规范，建成食品生产销售综合市场11个、农贸市场29个，创建餐饮食品安全示范街25条，被中国城市竞争力研究会评为“中国十佳食品安全城市”。深入化解社会矛盾，信访工作得到加强。推进社会治安综合治理，社会保持和谐稳定。国防建设、民族宗教工作扎实推进。统计、体育、地震、人防、气象、妇女儿童、残疾人等工作取得了新成绩。

积极转变政府职能，自身建设不断加强。严格落实改进工作作风、密切联系群众各项规定，集中开展正风肃纪活动，作风建设取得实效。自觉接受市人大法律监督、工作监督和政协民主监督，人大代表建议和政协委员提案全部办结。大力推进简政放权，市级行政审批事项由205项减为95项。充分发挥政务服务中心平台作用，扩大网上审批和电子监察范围。市级公共资源交易平台建成运行，政府采购、工程建设招投标、土地及矿产资源交易等实现管办分离、集中交易。政务公开、行政监察、审计监督和依法行政工作成效明显，反腐倡廉工作不断加强。

我们还承办了全国社会养老服务体系建设现场会、国家中西部农村危房改造现场会和全区社区服务与促进就业、重点区域绿化、食品安全经验交流会等一系列重要会议，农村养老、百姓安居、食品安全、就业创业、城乡绿化等工作成为全国、全区的典型示范模式。

各位代表!

过去一年，我们付出了艰辛的努力，取得了令人振奋的成绩。这些成绩的取得，是自治区党委、政府和市委科学决策、正确领导的结果，是我们立足市情，推进“8337”发展思路深入破题的结果，更是全市各族干部群众攻坚克难、团结奋斗的结果。在此，我代表市人民政府，向所有关心、支持和参与乌兰察布建设发展的同志们、朋友们，表示衷心的感谢并致以崇高的敬意!

成绩来之不易，经验弥足珍贵。回顾一年来的奋斗历程，我们深深体会到：实现科学发展、跨越发展，必须坚持解放思想、实事求是，既要敢闯敢试、先行先试，又要立足实际、步步为营。必须坚持抢抓机遇、顺势而为，突出发展主题，实现加快发展、转型发展、内涵发展。必须坚持问题导向、创新驱动，既要全力解决当前的棘手问题，更要积极化解深层次矛盾。必须坚持勇于负责、敢于担当，始终保持“人一我十、人十我百”的精神状态，推动全市经济社会又好又快发展。

在肯定成绩的同时，我们也清醒地看到，乌兰察布欠发达的市情还没有根本改变，主要表现在：经济总量小，产业层次低、链条短、结构单一，企业运行质量较差；基础设施承载能力不强，资源环境约束日益趋紧；群众关注的热点难点问题解决得还不够理想，发展环境不够优化，个别单位办事效率不高、服务意识不强的问题还不同程度存在。对于这些问题，我们要高度重视，认真加以解决。

二、2014年工作安排

2014年是贯彻落实十八届三中全会精神、全面深化改革的开局之年，也是实现“十二五”规划目标的攻坚之年。做好今年政府工作责任重大、意义深远，我们一定要解放思想，开拓创新，扎实推进改革发展各项工作。

今年政府工作的总体要求是：深入贯彻落实中央、自治区一系列重要会议和市委三届四次全委会议精神，始终坚持以“8337”发展思路统领全局，把改革创新贯穿于经济社会发展各领域各环节。以扩大有效投资为重点，确保经济快速增长；以扩张总量提升层次为核心，促进产业结构调整；以推进新型城镇化为龙头，加快城乡一体化发展进程；以增进人民群众福祉为目标，着力保障和改善民生，全面发展社会事业，推动经济社会健康协调发展，为全面建成小康社会奠定更加坚实的基础。

主要预期目标是：地区生产总值增长9.5%，财政公共预算收入增长12%，社会消费品零售总额增长12%，城镇居民人均可支配收入和农牧民人均纯收入分别增长10%和12%。

重点抓好十个方面的工作。

(一)促进投资稳定增长。充分发挥投资在稳增长方面的关键作用，在重点领域和关键环节实施一批大项目、好项目。固定资产投资完成944亿元，增长18%。

努力优化投资结构。瞄准国家政策和市场需求，在基础设施、生态环保、社会事业等领域加大项目争取力度，确保上级投资额度高于全区平均水平。扩大银行信贷投放和直接融资规模，中小企业、三农三牧贷款增速高于当年各项贷款平均增速，增量高于上年水平。突出企业投资主体地位，落实促进非公经济发展的各项政策措施，释放民间投资潜力，民间投资占全部投资比重达到60%以上。坚持把招商引资作为今后一个时期的头号工程，全面拓展一二三产招商领域，激活政府、市场、社会三个方面的力量，构建政府为主导，市场为主体，全社会广泛参与的招商引资格局。深入推进与京津晋冀等地的区域合作，加快察哈尔经济技术开发区和中关村、察右前旗和大兴区合作园区建设，探索园区共建、项目共管、收益共享的合作机制，年内确保落地开工一批重点项目。

加强重点领域投资。建设一批既拉动即期增长，又蓄积发展后劲的重大项目。全年实施亿元以上项目338个，完成投资817亿元；其中10亿元以上重大项目63个，完成投资240亿元。重点产业方面，实施亿元以上项目295个，当年完成投资694亿元。其中工业项目150个，完成投资390亿元；商贸物流项目43个，完成投资99亿元；房地产等其他产业项目102个，完成投资205亿元。道路交通方面，完成公路建设投资100亿元以上，重点推进“两纵四横一绕城”干线公路建设，开工科布尔至卓资、呼市至凉城等10个重点项目，中心城区绕城高速、G7高速建成通车，新建、续建高速公路355公里、一级公路609公里、二级公路827公里。建成农村牧区公路1000公里以上，嘎查村通沥青水泥路率达到75%，苏木乡镇客运站覆盖率达到94%。呼张客专全线开工，支线机场实现通航。电网建设方面，完成投资27.7亿元，实施化德南、黄旗海等34项110千伏以上输变电工程，推进重点工业园区电力扩容和农村牧区电网改造升级，新增变电容量519万千伏安。水利设施方面，完成投资12.8亿元，建成卓资隆盛等3座水库，规划建设吉庆峡等一批中小型水库，开工四子王旗银贵河等9座小(Ⅱ)型水库除险加固、察右前旗泉玉林河等6项中小河流治理工程。新增节水灌溉面积30万亩，解决15万人的饮水安全问题。

(二)努力提升农牧业现代化水平。打好生态牌、走好特色路，加快推进“绿色农畜产品生产加工输出基地”建设。

加强农畜产品基地建设。围绕“绿色、有机、安全”发展方向，重点提升“三个水平”，着力打造首都重要的绿色蔬菜供应基地、全国最大的马铃薯种薯生产供应基地和高效农业示范基地。提升集约化生产水平。以建设国家农业科技园区为抓手，推进现代农业科技创新和成果转化应用。以民丰、嘉恒等企业为龙头，打造马铃薯种薯联盟，种薯面积达到100万亩，生产合格种薯150万吨。以兴大业、绿蒙远大为重点，形成300万口商品仔猪和35万套种鸡的供种能力。以先进改良技术为支撑，完成杜蒙肉羊杂交30万只，优质良种肉羊比重达到90%以上。提升规模化种养水平。建设东起兴和西至四子王旗的马铃薯产业带，建成2—3处万亩以上集中连片马铃薯种植园区。设施蔬菜达到20万亩、冷凉蔬菜达到55万亩，打造草莓、樱桃、葡萄、食用菌等10个高标准特色产业园。抓好商都等5个旗县的百万口商品猪养殖，卓资等6个旗县的千万只肉鸡养殖，兴和、四子王旗的肉兔养殖。奶牛规模化养殖比例提高到60%以上。出栏生猪300万口、肉鸡2300万只、肉羊800万只、肉牛19万头。提升标准化生产水平。出台奖励扶持政策，建设马铃薯标准化生产基地80万亩、蔬菜标准化生产基地3.6万亩、“百千万”标准化养殖场30处。启动实施民丰薯业、卓资草莓等农业综合标准化示范区项目。认证无公害、绿色、有机农畜产品32个，新增认证面积8万亩。认证农畜产品地理标志3个，力争打造农畜产品“中国驰名商标”3件以上。完善农畜产品质量安全监管体系，创建2个安全监管示范县，建设市级检验检测中心。抓好动物疫病防控工作，确保不发生重大安全质量事故和区域性重大动物疫情。

培育壮大龙头企业。以薯、菜、乳、肉等产业为重点，引进、培育和扶持一批产业化龙头企业，推动农畜产品加工业集群化发展。设立市级农牧业发展资金5000万元，在贷款贴息、融资担保、资金补贴上给予支持。支持嘉恒、鹏程申报国家级农牧业产业化龙头企业，扶持民丰、中加等公司做大做强。规模以上农畜产品加工企业达到158家，加工转化率达到51%。

转变农牧业生产经营方式。建立市县乡三级土地、草牧场流转服务机构和信息平台，鼓励土地、草牧场向专业大户、家庭农牧场、专业合作社和龙头企业流转，流转面积分别新增100万亩。推进专业合作示范社建设，评定市级示范社100家，争创自治区级示范社30个、国家级示范社5个。推广“龙头企业+合作社+农户”合作模式，提高农牧业生产机械化和市场组织化水平。

(三)做大做强工业经济。坚持延伸与发展同步，升级与集聚并重，全面提升产业综合竞争力。规模以上工业增加值达到405亿元，增长14%。完成工业固定资产投资475亿元，增长18%。

大力扩张产业规模。推进规模化、多元化、长链条发展，引进建设一批重点项目。加快能源基地建设。新增火电装机100万千瓦、风电装机100万千瓦、太阳能发电装机40万千瓦，打造千万级电力能源基地。重点推进集宁热电2×35万千瓦、岱电三期2×66万千瓦等5个火电项目，加快化德北辰等4个自备电厂建设。争取尽快启动乌兰察布至武汉特高压电力外送通道建设。抓好呼兴天然气管道建设，加快卓资、兴和天然气液化项目建设。推动天津渤海化工、天津能源、北京燃气集团煤制天然气项目落地开工。加快四子王旗等地煤田开发进度。延伸化工产业链条。氟化工系列产品达到21个，综合产能达到64万吨。推进三爱富、万豪、奥特普3家企业股权重组，加快以万吨聚偏氟乙烯为主的系列配套项目建设。四子王旗永和12万吨氟化工系列产品项目建成投产、新型制冷剂项目开工建设。石墨碳素化工系列产品超过100个，产能达到27万吨。重点推动投资28亿元的瑞盛二期石墨深加工项目开工建设。抓好三信实业4万吨超高功率石墨电极项目和50兆瓦太阳能电站项目建设。力争海霸能源10亿安时动力锂电池、木子碳素5000吨等静压石墨等项目竣工投产。电石化工及下游产品综合产能达到490万吨。重点抓好投资43亿元的蒙维二期聚乙烯醇及下游延伸项目，推进10万吨PVA和特种纤维项目建设，打造国内重要的PVA生产基地。佳辉硅化工8万吨有机硅深加工项目投产运营，拉动四子王旗20万吨工业硅项目满负荷生产。

努力提升产业层次。坚持传统产业新型化、新兴产业规模化、支柱产业多元化，推动工业经济优化升级。

设立5000万元市级工业发展资金，支持企业技术创新，开发优势明显、适销对路、节能环保的新产品，提高市场竞争力。加快传统产业改造升级。坚守产业政策和环保达标两条底线，利用市场和政策双重机制，倒逼现有电石和铁合金企业技改升级、节能降耗，调整产品结构，加快兼并重组。坚持“上大压小、等量置换”，加快淘汰小容量、开放式、高污染矿热炉，新上项目必须达到大容量、规模化、高水平，同步配套节能环保设施。冶金产业要向标准件铸造、精密合金冶炼、型材加工和不锈钢终端产品制造延伸。投资30亿元的丰镇亚泰百万吨镍铬合金项目一期工程建成投产。大力发展新兴产业。推动电石化工向高档涂料、高强纤维等系列终端产品发展，氟化工向高性能橡胶系列产品发展，硅化工向有机硅、树脂、光伏材料系列产品发展。石墨碳素化工向石墨烯、金刚石、石墨纸等高端产品发展，推进瑞盛能源与清华大学深度合作，采用石墨关键应用技术，开发新产品。加大生物制药和保健系列饮品的开发力度，福瑞健康科技园项目年内竣工。积极推动华为云计算项目落地，引进电子商务和物联网等企业，建设“智慧城市”。

加快建设特色园区。坚持统一规划、合理布局、突出特色，错位发展，加快园区提档升级步伐。加大重点园区基础设施建设力度，完善配套功能，提升管理和服务水平，增强产业承载能力。以丰镇氟化工园区为依托，加大项目引进力度，实现产品集聚配套，产业集群发展，打造全国重要的精细氟化工产业基地。抓好兴和石墨碳素、集宁皮革、化德羊驼绒服装、四子王旗石材等特色园区建设。争取丰镇氟化工园区、后旗建材化工园区升级为自治区级园区和自治区产业示范基地，推动丰镇氟化工园区和卓资旗下营工业园区分别列入国家、自治区循环经济试点园区。

(四)加快现代服务业发展步伐。坚持生产性服务业与生活性服务业并重，现代服务业和传统服务业并举，实现发展提速、比重提高、水平提升。第三产业增加值增长13%。

不断壮大现代物流业。围绕建设区域性商贸物流中心，完善现代物流发展体系，提升重点园区综合服务功能，提高物流业信息化、现代化水平。发挥海关作用，加强口岸合作，推进“内陆港”和“保税区”设立工作。“三个交易中心”实现营业收入165亿元。煤炭交易中心，以西北煤炭电子交易信息平台建设为载体，推动煤炭产销平台建设和营销方式创新，启动运营博源煤炭洗选加工和伊泰铁路货场。煤炭吞吐量达到2000万吨，实现营业收入100亿元。马铃薯交易中心，以聚源等企业为重点，推进马铃薯产地批发市场建设；以嘉恒农科北京新发地批发市场周转库建设为重点，推进京津地区农产品销地集配中心建设；以供应北京高校联合采购中心为突破口，深化“农批、农超、农校”对接。马铃薯年交易量达到300万吨，实现营业收入50亿元。皮革交易中心，建立以集宁国际皮革城为主的销售中心、以鑫源通皮件产业园为主的加工基地、以土牧尔台为主的制革基地，形成集鞣制、加工、销售于一体的完整产业链。争取设立国家级皮革研发和质检中心，推动皮革产业创新和质量提升。皮件营业收入突破15亿元。

大力发展文化旅游业。围绕打造“体现草原文化、独具北疆特色的旅游观光休闲度假基地”，全面推进“一心两翼三大境区”建设，打响“吉祥草原、避暑胜地”品牌。推进精品旅游景区建设。引进有实力的大型旅游企业或管理公司，加大重点景区整合和建设力度，格根塔拉、岱海旅游区创建5A级景区，辉腾锡勒创建4A级景区。促进文化旅游融合发展。深入挖掘察哈尔文化、草原文化、红色文化资源，加快建设一批文化旅游名城名镇名村。重点推进元代集宁路遗址博物馆、集宁古城、凉城绥蒙革命纪念园等项目建设。乌兰花镇争创全国旅游名镇，隆盛庄镇争创全国历史文化名镇。提升服务水平、推动区域合作。推进旅游业服务标准化试点建设，提升旅游景区经营服务水平。设立市县两级旅游发展专项资金。建成乌兰察布游客服务中心，深化与周边城市的合作，积极拓展客源市场。全年接待游客达到900万人次，实现旅游收入100亿元以上。

积极发展商贸流通业。加快培育市场体系，拓展流通方式，完善配套功能。重点推进数码大厦、国际商贸城、维多利城市广场等商贸综合体项目建设。抓好“新网工程”、“万村千乡市场工程”，完善社区农畜产品销售网络，中心城区改造20家、每个旗县改造5家销售网点。加强粮食储备、流通和市场体系建设。大力发展连锁经营、电子商务、物流配送等新型业态，提升商贸流通现代化、信息化水平。

加快发展现代金融业。完善促进金融业发展政策措施，引进股份制银行和民营中小金融机构。推进农信社股份制改革，乌兰察布农村商业银行挂牌运营。推广化德“羊驼绒通宝”、集宁“政银保”等融资模式，市本级设立3000万元专项资金作为“蓄水池”，最大限度撬动银行贷款，满足中小微企业融资需求。推进社会信用体系建设，规范发展民间投融资机构，防控地方金融风险。

(五)加快推进新型城镇化。抓住国家加快新型城镇化进程的有利机遇，全面提高城镇化发展质量和水平。完成城镇建设投资265亿元，城镇化率达到46%以上。

提高城镇规划水平。坚持让人民群众生活更舒适的规划理念，科学设置开发强度，合理划定开发边界，优化城镇布局设计，避免千篇一律、千城一面。中心城区完成总体规划实施评估工作，在全面完成旗县市新一轮总体规划修编的基础上，深度编制重点区域规划和城市设计，实现所有建制镇总规、控制性详规和专项规划全覆盖。编制完成300个以上村庄规划。

提高城镇建设水平。坚持科学发展、理性建设，加快城市内涵发展步伐，促进中心城区、县城和建制镇协调发展。打造宜居城市品牌。中心城区进一步完善“一市两区三组团”发展架构，统筹抓好城市微观空间治理，提高城市精细化建设水平。加强基础设施和公共服务体系建设，进一步增加文化内涵，不断完善城市功能。以“三山两河”为重点，拓展绿化空间，新增城市绿地面积3万亩，争创国家园林城市。旗县市以扩容提质为重点，统筹推进城关镇和重点镇建设，打造规模适度、功能完善、环境优美、宜居宜业的特色城镇。凉城等4个旗县市争创自治区园林县城。完善市政设施功能。全市实施市政基础设施建设项目300个，完成投资100亿元。中心城区完成基础设施投资31亿元，开展地下管网普查工作，推进综合管廊设施建设，中水回用率、垃圾集中处理率和燃气普及率分别达到87.5%、98.5%和63%。各旗县市供水供热、市政道路、垃圾处理、电力通讯等基础设施建设，达到自治区平均水平。提升住宅小区品质。全市改造老旧住宅小区40个，完成既有建筑节能改造200万平方米，绿色建筑占新增建筑总量的15%。大力推广全装修高品质住宅小区，商品房竣工面积350万平方米。开展物业管理示范创建活动，物业管理覆盖率提高到50%以上。

提高城市管理水平。建立多元可持续的资金保障机制，鼓励社会资本参与城市公用设施投资运营。加强城市网格化管理。加大市容市貌综合治理力度，重点抓好城市出入口环境集中整治。

统筹推进常年在城镇稳定就业、生活的农牧民实现市民化。

(六)不断加强生态文明建设。牢固树立生态文明理念，严格落实主体功能区规划，实现美丽与发展双赢。

加强生态工程建设。创新绿化投入机制，实施京津风沙源治理等国家重点生态项目，推进绕城高速、道路节点及出入口等重点区域绿化，开展园区、社区、机关、学校、村庄“五个一”绿色家园创建活动。完成营造林152万亩，建设人工草地300万亩。创新经营管护机制，加大禁牧力度，巩固生态建设成果。深化林权制度改革，促进林地林木依法流转。创新产业发展机制，加强种苗基地建设，新增苗圃面积3万亩，提高林木种苗自给率。每个旗县引进培育2-3家大型加工企业，推动林草资源向林草产业转变。

加大环境保护力度。加强大气污染防治工作，严格环境准入，兴和、商都不再审批电石、铁合金、焦化等新增产能项目。加快重点行业污染防治，电力等重点工程全面完成脱硫、脱硝改造。完善工业园区环保基础设施，确保各类污染物达标排放。加强机动车污染检测，实施“黄标车”区域限行和强制淘汰政策。建成中心城区PM2.5监测站并发布空气质量监测信息。加强城镇污水处理厂改造建设，城镇污水处理率提高20%以上。圆满完成4项主要污染物年度减排任务。

推进资源节约集约利用。切实提高建设用地利用率，加大废弃居民点和工矿废弃地复垦项目实施力度，促进国土资源节约和高效利用。争取自治区延续地勘扶持政策，推进合作勘查和整装勘查，实施100个以上地勘项目。进一步规范矿业权设置，促进资源就地转化和深加工。

(七)努力保障和改善民生。统筹做好各项民生工作，努力使更多人民群众得到实惠。

推进扶贫攻坚工程。紧紧围绕“两不愁、三保障”目标，深入实施“三三制”扶贫战略。重点抓好基础建设、公共服务、特色产业、村容村貌、生态环境和基层组织建设六项工作，实现贫困嘎查村和贫困户“五通、八有、一产业、一项目”。在工作摆布上，继续推进领导干部联系扶贫点机制，扎实开展规划、项目、干部“三到村三到户”工作，全面启动500个贫困嘎查村的整村推进工作，年内完成132个重点贫困嘎查村整村推进、4000户移民搬迁任务。在资金投入上，加大涉农涉牧资金整合力度，发挥财政资金杠杆作用，投入1.8亿元财政担保资金，撬动18亿元扶贫惠民贷款，增强贫困对象的自我发展能力。在扶贫对象上，建立精准扶贫机制，开展农村牧区贫困户建档立卡、动态管理工作，提高扶贫工作的针对性。年内实现10万以上贫困人口稳定脱贫。

推进就业创业工程。千方百计稳定和扩大就业，深入实施就业“五项工程”。大力推广“乌兰美”培训模式，依托院校搞培训，面向公司促就业，每个旗县市区打造1个劳务品牌，每所职业学校打造1个培训品牌。加强标准化实训基地建设，围绕重点产业和企业用工需求，建成6个市级实训基地，打造3个自治区标准化实训基地。城镇新增就业1.6万人，转移城乡劳动力70万人。

推进百姓安居工程。围绕“住有所居、居有改善”的目标，加大保障性住房建设力度，建设城镇保障性住房5.5万套。加快“美丽乡村”建设，推广四子王旗农危改的经验做法，统筹推进农村牧区危房改造、村容村貌整治和新农村新牧区建设。年内完成G6等16条公路和京包、集通铁路沿线的村庄整治任务。继续推进农村养老互助幸福院建设，进一步完善配套设施，提高管理水平。

完善社会保障体系。提高医疗保障水平，城镇基本医疗保险覆盖率达到98%，城镇居民医疗保险最高支付限额由13万元提高到20万元，新农合筹资标准由340元提高到390元，门诊统筹人均由60元提高到80元，将恶性肿瘤全部纳入重大疾病报销范围。完善异地参保报销办法，在呼市、包头设立社会保险和新农合服务网点。进一步扩大城镇职工基本养老保险覆盖面，制定基本养老保险关系转移接续办法。推进养老服务社会化，实现信息服务全覆盖。提高城乡低保和五保对象保障标准，城镇低保月保障标准提高到450元，农村牧区低保年保障标准提高到3180元，五保集中供养和分散供养标准分别提高到7300元、3500元。

推进社会管理创新。加强社会治安防控体系建设，推进社区网格化管理，加强视频监控建设，依法打击各类违法犯罪活动，保持治安形势稳定。完善应急管理机制，提高预防和应对突发公共事件能力。加大安全生产隐患排查力度，建立安全生产监管长效机制。加强和改进信访工作，加大矛盾纠纷排查化解力度。深入开展“六五”普法。加强人防、地震等工作。抓好国防教育和双拥工作，巩固发展军政军民团结。

(八)全面发展社会事业

优先发展教育事业。深入推进“三项改革”，抓好师德和教师能力提升“两项工作”，着力提高教育教学质量。推进义务教育均衡发展，实施“名校带新校、名校带弱校、城市带农村”工程，促进学校内涵式发展。加大学前教育普及力度，抓好幼儿园运行管理，学前三年入园率由50%提高到70%。提高职业教育创新水平，组建职业教育集团，打造职业教育品牌。引导和支持民办教育标准化办学，加快民族教育、特殊教育、高等教育发展步伐。推进北京八中分校品牌创建，集宁师院附中、凉城一中创建自治区示范高中。

大力发展医疗卫生事业。按照“保基本、强基层、建机制”的要求，深化医疗卫生体制改革。以提高医疗管理服务水平为重点，加强医疗机构内涵建设，严格控制医药费用不合理增长。推进市中心医院等4所市县医院的等级创建工作，完成2所县级医院、14所乡镇卫生院和80所村卫生室标准化建设。建立并完善市县两级120急救指挥平台。加强医疗卫生基础设施建设，确保市卫生园区一期工程投入使用，抓好市第二医院、市妇幼保健院等项目。完善基本公共卫生服务体系。启动食品药品监管机构改革，加强食品药品质量安全监管，建立从生产加工到流通消费的全程监管制度，严厉打击违法违规行为，确保人民群众“舌尖上的安全”。

加快推进文化等社会事业。着力提高公共文化服务水平，推进博物馆、科技馆建设，实施6万户广播电视“户户通”等惠民工程。加快文化产业发展步伐，抓好元代集宁路文化产业园等重点项目，文化产业增加值突破10亿元。保持生育水平总体稳定，提高出生人口素质。深入开展全民健身活动，备战十三运会。加强科技创新工作，加大示范推广力度，提高服务发展能力。全面落实中长期人才发展规划，完善人才公共服务平台，加大人才培养力度。加强民族宗教工作。统筹发展妇女、儿童、老龄和残疾人事业。

(九)深化重点领域和关键环节改革

推进财税体制改革。建立全口径政府预算体系，提高预算编制的完整性、准确性。加强非税收入收缴管理，将非税收入全额纳入预算。加强财政支出管理，完善国库集中支付、财政投资评审管理制度。加强政府债务管理，积极化解现有债务，合理举借新的债务，建立负债有度、用债有方、管债有规、举债有责的管理机制。规范转移支付制度，完善县级基本财力保障机制。

进一步激发市场主体活力。废除对非公有制经济各种不合理规定，消除隐性壁垒。深化市场准入制度改革，推进“宽进严管”的监管制度，为市场主体提供便捷式服务。整顿规范财税、土地、资源配置等优惠政策，建立公平开放透明的市场规则。

健全城乡一体化发展体制机制。完善农村牧区土地产权制度，建立城乡统一的建设用地定价评估体系，推进城乡要素平等交换和公共资源均衡配置。稳步推进“扩权强县”改革，不断增强县域经济发展活力。

(十)切实加强政府自身建设

着力转变政府职能。正确处理市场与政府的关系，规范政府行为，提高政府治理能力。减少政府对微观经济活动的干预，把更多精力放在改善民生和提高社会公共服务水平上。做好行政审批权的“接、放、管”工作，接好上级下放的审批事项，把本级该放的权力放到位，把政府该管的事项管理好。市级行政审批事项由95项减为83项。加快政务服务中心标准化建设，实现审批单位人员和项目100%进驻中心，驻厅服务窗口与主管单位100%联网，政务服务电子监察100%覆盖。

着力推进依法行政。自觉接受人大及其常委会监督，积极支持人民政协履行职能，认真办理人大代表建议和政协委员提案。推进政府工作法制化，畅通行政复议渠道，化解行政争议，强化行政监督，规范行政行为。大力推进政务公开，不断提高政府的公信力和执行力。

着力加强作风建设。围绕“为民务实清廉”，深入开展党的群众路线教育实践活动。坚决贯彻中央改进工作作风八项规定、自治区二十八项配套规定和我市具体办法，认真落实精简会议、改进调查研究、规范公务接待等要求，集中整治干部队伍中存在的“四风”问题，着力提高行政效能和群众满意度。

着力强化廉政建设。深入推进廉政风险防控管理，全面落实廉政建设责任制，严格执行“三重一大”各项规定，从源头上预防和治理腐败。强化行政监察和审计监督，开展工程建设、政府采购等重点领域突出问题专项治理。深化公共资源交易制度改革，逐步建立统一规范的公共资源交易市场。加强机构编制、公务用车、办公用房等工作的规范管理，严格控制“三公”经费，反对奢侈浪费，厉行勤俭节约，真正做到“政府过紧日子、人民过好日子”。

各位代表，让乌兰察布更美好，让人民群众更幸福，始终是我们一切工作的出发点和落脚点。今年市政府郑重承诺，要继续为民办好十件实事。

一是对自治区贫困线以下、不具备生产条件和丧失劳动能力的农牧民，发放1500元现金补贴。

二是为全市32万户低收入农牧民每户提供1吨取暖用煤。

三是提高大病患者的医疗费用报销比例和医疗救助水平。城镇职工当年医疗费用总额超过5万元的、城镇居民和参合农牧民当年医疗费用总额超过3万元的，属于政策范围内的个人自付部分再报销50%。同时对其中符合医疗救助条件的低收入群体，在上述报销比例的基础上由民政部门再救助10%。

四是对当年新入学的3000名普通高等专科以上农村牧区贫困家庭大学生，在校期间每人每年救助1500元；对当年新入学的1000名普通高等专科以上城市低保家庭大学生每人一次性救助3000元。

五是对已经在教育主管部门备案、纳入统一管理的农村牧区幼儿园，实行幼儿免费入园。

六是把2014年作为“社区场所建设年”，完成全市59个300平方米以下社区综合服务场所改造，其中新建场所面积达到650平方米以上。

七是实现中心城区重点餐饮单位食品安全电子追溯和重点药品零售企业在线监控。每个旗县市区新创建食品安全示范街1条、示范学校食堂1家，食品生产加工、销售、餐饮示范店和药品示范店各5家。

八是对全市2175户农村牧区危土窑全部拆除并新建砖瓦房，完成2.5万户以上农村牧区危房改造。

九是中心城区及旗县市城关镇小街小巷硬化、亮化覆盖率由80%提高到90%以上。

十是在农村牧区布设惠农取款服务网点500个，解决农牧民取款难、用卡难问题。

各位代表!百舸争流，破浪者领航；千帆竞发，奋勇者当先。乌兰察布已步入了跨越发展的关键时期，新形势催人奋进，新征程任重道远。让我们在市委的坚强领导下，解放思想，锐意进取，攻坚克难，扎实工作，为全面建成小康社会而努力奋斗!
